# Angiotensin-Converting Enzyme Inhibitor Protects Against Cisplatin Nephrotoxicity by Modulating Kinin B1 Receptor Expression and Aminopeptidase P Activity in Mice

**DOI:** 10.3389/fmolb.2020.00096

**Published:** 2020-05-20

**Authors:** Gabriel R. Estrela, Frederick Wasinski, Marcos F. Gregnani, Leandro C. Freitas-Lima, Adriano C. Arruda, Rafael Leite Morais, Denise MAC Malheiros, Niels O. S. Camara, João Bosco Pesquero, Michael Bader, Carlos Castilho Barros, Ronaldo Carvalho Araújo

**Affiliations:** ^1^Departamento de Medicina, Disciplina de Nefrologia, Universidade Federal de São Paulo, São Paulo, Brazil; ^2^Departamento de Oncologia Clínica e Experimental, Disciplina de Hematologia e Hematoterapia, Universidade Federal de São Paulo, São Paulo, Brazil; ^3^Departamento de Imunologia, Instituto de Ciências Biomédicas, Universidade de São Paulo, São Paulo, Brazil; ^4^Departamento de Biofísica, Universidade Federal de São Paulo, São Paulo, Brazil; ^5^Departamento de Clínica Médica, Universidade de São Paulo, São Paulo, Brazil; ^6^Max-Delbrück Center for Molecular Medicine (MDC), Berlin, Germany; ^7^Institute for Biology, University of Lübeck, Lübeck, Germany; ^8^Charité University Medicine, Berlin, Germany; ^9^German Center for Cardiovascular Research (DZHK), Partner Site Berlin, Berlin, Germany; ^10^Departamento de Nutrição, Escola de Nutrição, Universidade Federal de Pelotas, Pelotas, Brazil

**Keywords:** cisplatin nephrotoxicity, kinins, aminopeptidase P, inflammation, enalapril

## Abstract

Cisplatin is a highly effective chemotherapeutic agent. However, its use is limited by nephrotoxicity. Enalapril is an angiotensin I-converting enzyme inhibitor used for the treatment of hypertension, mainly through the reduction of angiotensin II formation, but also through the increase of kinins half-life. Kinin B1 receptor is associated with inflammation and migration of immune cells into the injured tissue. We have previously shown that the deletion or blockage of kinin B1 and B2 receptors can attenuate cisplatin nephrotoxicity. In this study, we tested enalapril treatment as a tool to prevent cisplatin nephrotoxicity. Male C57Bl/6 mice were divided into 3 groups: control group; cisplatin (20 mg/kg i.p) group; and enalapril (1.5 mg;kg i.p) + cisplatin group. The animals were treated with a single dose of cisplatin and euthanized after 96 h. Enalapril was able to attenuate cisplatin-induced increase in creatinine and urea, and to reduce tubular injury and upregulation of apoptosis-related genes, as well as inflammatory cytokines in circulation and kidney. The upregulation of B1 receptor was blocked in enalapril + cisplatin group. Carboxypeptidase M expression, which generates B1 receptor agonists, is blunted by cisplatin + enalapril treatment. The activity of aminopeptidase P, a secondary key enzyme able to degrade kinins, is restored by enalapril treatment. These findings were confirmed in mouse renal epithelial tubular cells, in which enalaprilat (5 μM) was capable of decreasing tubular injury and inflammatory markers. We treated mouse renal epithelial tubular cells with cisplatin (100 μM), cisplatin+enalaprilat and cisplatin+enalaprilat+apstatin (10 μM). The results showed that cisplatin alone decreases cell viability, cisplatin plus enalaprilat is able to restore cell viability, and cisplatin plus enalaprilat and apstatin decreases cell viability. In the present study, we demonstrated that enalapril prevents cisplatin nephrotoxicity mainly by preventing the upregulation of B1 receptor and carboxypeptidase M and the increased concentrations of kinin peptides through aminopeptidase activity restoration.

## Introduction

Cisplatin is an effective antitumor agent often used in chemotherapy to treat multiple types of tumor. However, cisplatin nephrotoxicity is a limiting factor for its use (Arany and Safirstein, 2003; Miller et al., 2010). In order to protect the kidney against injury, it is necessary to understand the relationship between renal injury and several signaling pathways, and how this results in necrosis and apoptosis of renal tubular cells. It is well-described that during cisplatin treatment, immune cells and pro-inflammatory cytokines are increased in the kidney (Okusa, 2002; Ramesh and Reeves, 2002, 2003; Miller et al., 2010).

Enalapril is a highly effective angiotensin-converting enzyme (ACE) inhibitor and is widely used as an antihypertensive drug. It acts not only by preventing the formation of angiotensin II (AngII), which is the main effector vasoconstrictor peptide of the renin-angiotensin system (RAS), but also by avoiding the degradation of vasodilator peptides of the kallikrein-kinin system (KKS) (Marcic and Erdös, 2000; Souza Dos Santos et al., 2001). The main effect of enalapril in the control of hypertension is related to the inhibition of AngII formation. This peptide has shown to be able to activate inflammatory genes regulated by NF-κB, both *in vivo* and *in vitro* (Passos-Silva et al., 2015).

Kinins are well-known for their ability to affect immune response. Our group has already shown the role of both kinin receptors in cisplatin-induced acute kidney injury, where the deletion or blockage of kinin B1 and B2 receptors attenuates the nephrotoxicity caused by cisplatin exposure (Estrela et al., 2014a,b). Studies have revealed that ACE influences the regulation of both kinin receptors in vascular smooth muscle cells (Ignjacev-Lazich et al., 2005). Moreover, it has already been shown that ACE inhibition protects against some types of renal disease (Ghosh et al., 2009, 2012; Vejakama et al., 2012; Ding et al., 2014; Ham et al., 2018; Panico et al., 2019), including cisplatin-induced kidney disfunction (El-Sayed el et al., 2008), but only renal function and reactive oxygen species were assessed in this work. Given that (a) inflammation has an important role in cisplatin nephrotoxicity; (b) AngII influences the regulation of inflammation-related genes; and (c) ACE inhibition reduces kinin degradation, we decided to verify whether the protective effect of ACE inhibition on the attenuation of cisplatin nephrotoxicity is related to KKS regulation.

## Materials and Methods

### Animals

Male C57BL/6 mice weighing 25–30 g and aged 12–14 weeks were used for these experiments. The animals were obtained from the Animal Care Facility of the Federal University of São Paulo (UNIFESP). All animals were housed in individual, standard cages and had free access to water and food. All procedures were previously reviewed and approved by the internal ethical committee of the Federal University of São Paulo (CEUA 155783).

### Experimental Protocol

The mice were divided into 3 groups for each experiment: control group, cisplatin (CIS)-treated group and cisplatin plus enalapril (ENAL+CIS)-treated group. We used *n* = 5–6 for each experiment and condition.

### Drug Treatment

Single doses of Cisplatin (Ramesh and Reeves, 2002, 2003; Estrela et al., 2014b, 2017a,b) (20 mg/kg—Bergamo, Taboão da Serra, Brazil) were injected intraperitoneally. Tissues and blood were collected 4 days after injection. Enalapril (1.5 mg/kg i.p) < (Cozzoli et al., 2011; Jackson et al., 2013; Fendrich et al., 2014) > was dissolved in PBS and the treatment started 1 day prior to cisplatin administration and was given daily until tissue collection. Control animals received PBS intraperitoneally at same volume as cisplatin.

### Blood Sampling and Tissue Collection

The mice were anesthetized with ketamin (91 mg/kg) and xylazin (9.1 mg/kg) intraperitoneally, and blood was collected via heart puncture. Blood was allowed to clot for 2 h at room temperature and then centrifuged for 20 min at 2,000 x g. The samples were then stored at −20°C. Kidney tissue was collected and renal capsule was removed. Transversal cuts were performed and the kidneys were immediately frozen in nitrogen and then stored at −80°C.

### Renal Function

Serum creatinine and urea levels were used to determine renal function. Samples were analyzed using commercially available colorimetric assay (Pereira et al., 2011; Estrela et al., 2014a,b, 2017a) kits (Labtest, Lagoa Santa, Brazil).

### Quantification of Gene Expression

Kidney samples were frozen at −80°C immediately after collection. Total RNA was isolated using TRIzol Reagent (Invitrogen, Carlsbad, CA). The RNA integrity was assessed by electrophoresis on an agarose gel. cDNA was synthesized using the “High Capacity cDNA Reverse Transcription Kit” (Applied Biosystems). Standard curves were plotted to determine the amplification efficiency for each primer pair. Real-time PCR was performed using two systems: the TaqMan system (Applied Biosystems, Carlsbad, CA) using probes for IL-6 (mm00446190-m1), TNF-α (mm00443258-m1), B1R (mm00432059-s1), B2R (mm00437788-s1) and GAPDH (mm99999915-g1); and the SYBR Green system (Thermo Scientific, Waltham, MA) using specific primers for β-actin, IL-1β, NGAL, Bax, Bcl-2, Tnfr-2, Ace, AT1R, AT2R, and CPM; the primers were designed using primer3 web and their specificity was confirmed using NCBI primer-BLAST; their sequences are shown in Table 1. The cycling conditions for both TaqMan and SYBR Green reactions were as follows: 10 min at 95°C, followed by 45 cycles of 30 s at 95°C, 30 s at 60°C, and 30 s at 72°C. Target mRNA expression was normalized to β-actin for SYBR and to GAPDH for TaqMan, and expressed as a relative value using the comparative threshold cycle (Ct) method (2–ΔΔCt) (Livak and Schmittgen, 2001). The expression levels of the genes of interest were normalized to the control group and presented as fold change.

**Table 1:** Sequences of primers used in real-time PCR assays.

**Gene**	**Primers**
β-actin	5′-CTGGCCTCACTGTCCACCTT-3′ 5′-CGGACTCATCGTACTCCTGCTT-3′
IL-1β	5′-AGGAGAACCAAGCAACGACA-3′ 5′-CGTTTTTCCATCTTCTTCTTTG-3′
BAX	5′-CGGCGAATTGGAGATGAACTG-3′ 5′-GCAAAGTAGAAGAGGGCAACC-3′
BCL-2	5′-ACCGTCGTGACTTCGCAGAG-3′ 5′-GGTGTGCAGATGCCGGTTCA-3′
TNFR-2	5′-GTCGCGCTGGTCTTCGAACTG-3′ 5′-GGTATACATGCTTGCCTCACAGTC-3′
ACE	5′-CTCAGCCTGGGACTTCTACAAC-3′ 5′-CTCCATGTTCACAGAGGTACACT-3′
AT1R	5′-CCATTGTCCACCCGATGAAG-3′ 5′-TGCAGGTGACTTTGGCCAC-3′
AT2R	5′-CAGCAGCCGTCCTTTTGATAA-3′ 5′-TTATCTGATGGTTTGTGTGAGCAA-3′
NGAL	5′-ATGTGCAAGTGGCCACCACG-3′ 5′-CGCATCCCAGTCAGCCACAC-3′

*IL-1β, interleukin 1β; BAX, Bcl-2-associated X; BCL-2, B-cell lymphoma 2; TNFR-2, tumor necrosis factor receptor 2; ACE, angiotensin converting enzyme; AT1R, angiotensin II receptor type I; AT2R, angiotensin II receptor type II. NGAL, neutrophil gelatinase-associated lipocalin*.

### Histological Analyses

Formaldehyde-fixed paraffin sections of kidneys were stained with hematoxylin and eosin (H&E). Optic light microscopy was employed to analyze the samples. Images were acquired at ×40 magnification. Epithelial desquamation, cellular debris, epithelial flattening, presence of cylinders, and dilation of the tubular lumen were used as criteria for tubular injury. The injuries were graded using a scoring procedure, in which grade 1 = 0–5% of the total kidney area was compromised; grade 2 = 6–10%; grade 3 = 11–25%; grade 4 = 26–50%, and grade 5 ≥ 50% (Estrela et al., 2014a,b, 2017a; Barrera-Chimal et al., 2017). Histological analysis was performed blind to experimental groups.

### Enzyme-Linked Immunosorbent Assay

Serum samples were frozen and stored at −20°C immediately after collection. Serum levels of TNF-α (MTA00B), IL-1β (MLB00C) and IL-6 (M6000B) were quantified using Quantikine ELISA mouse kits specific for each cytokine (R&D Systems, Minneapolis, MN), according to the manufacturer's instructions.

### Aminopeptidase P Activity

The assessment was performed by fluorescence measurement (λex = 340 nm and λem = 410 nm) followed by hydrolysis of specific substrate Lys(Abz)-Pro-Pro-pNA (Bachem). Briefly, an aliquot of homogenized tissue or mouse serum was incubated with buffer (0.1 M Tris-HCl, 0.5 mM MnCl_2_, pH 8.0) and 20 μM of Lys(Abz)-Pro-Pro-pNA substrate at 37°C, under stirring, for 20 min (Abid et al., 2009). The fluorescence was detected and analyzed by spectrofluorometer (SpectraMax Gemini XS, Molecular Devices). The fluorescence parameter was set as arbitrary fluorescence units. Apstatin was used as inhibitor for Aminopeptidase P activity at 10 μM.

### Cell Treatments

Mouse epithelial tubular cells MM55.K (ATCC) were cultured in Dulbecco's modified Eagle's medium high (DMEM high; *ThermoFisher*), 10% fetal bovine serum (FBS; *Dutcher*), and 1% Penicillin Streptomycin (P/S; *ThermoFisher*). The cells were incubated at 37°C with 5% CO_2_. At 90% confluence, they were exposed to 0.05% trypsin-EDTA (*ThermoFisher*) and seeded in 6-well plates with 0.1 × 10^6^ cells per well for qPCR and 96-well plates with 2,000 cells per well for MTT assay. The cells were incubated for 24 h with Enalaprilat (5 μM, *Sigma*) or Apstatin (10 μM, *Enzo Life Science*), both diluted in H_2_O and thereafter treated or not for 24 h with cis-Diammineplatinum(II) dichloride (50 or 100 μM, *Sigma*), diluted in 0.9% NaCl solution.

### Cell Viability Assay

After treatments, cell viability was measured with a cell proliferation kit (MTT, *Sigma*) according to the manufacturer's instructions. Briefly, MTT labeling reagent was added to the cell culture media for 4 h at 37°C, and then the solubilization solution was added overnight at 37°C. The absorbance values were obtained at 550 nm with Infinite 200 Pro (*Tecan*).

### Statistical Analysis

All data is presented as mean ± s.e.m. Intergroup differences significance was assessed by one-way analysis of variance (ANOVA) with the Tukey correction for multiple comparisons. The value for statistical significance was established at P < 0.05. All statistical analyses were performed using GraphPad Prism 5 (GraphPad, La Jolla, CA).

## Results

### Enalapril Reduces Cisplatin-Induced Renal Injury

**Figure 1:**
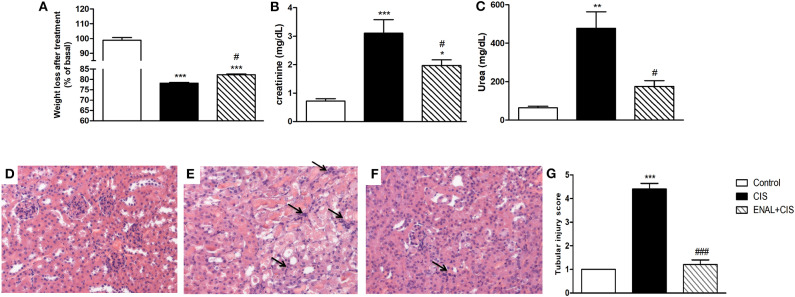
Effect of enalapril treatment in renal injury and function. Male mice were treated with cisplatin (CIS) or cisplatin plus enalapril (ENAL+CIS). **(A)** After 4 days all cisplatin-treated groups had weight loss; Both serum creatinine and urea levels were reduced by enalapril treatment compared to CIS group. **(B,C)** Tubular injury score analyzed by histology was smaller in ENAL+CIS group compared to CIS group **(D–G)**. **(D)** Control **(E)** CIS **(F)** ENAL+CIS. Arrows indicates inflammatory infiltration *p < 0.05, **p < 0.01, ***p < 0.001. compared to the control group. #*p* < 0.05; ###*p* <0.001. compared to the CIS group. Data presented as Mean ± SEM *n* = 5–6.

We injected enalapril (1.5 mg/kg i.p) as a preventive treatment in mice treated with cisplatin (20 mg/kg i.p). All animals treated with cisplatin presented weight loss, but enalapril was able to attenuate this effect (Figure 1A). We measured renal function markers and enalapril-treated mice presented lower creatinine and urea serum levels (Figures 1B,C). Moreover, enalapril-treated mice (ENAL+CIS group) showed lower tubular injury scores as analyzed by histology, presenting less epithelial desquamation, cellular debris, epithelial flattening, presence of cylinders, and dilation of the tubular lumen than animals treated with cisplatin alone (CIS group) (Figures 1D–G).

### Inflammatory Cytokines and Apoptosis Markers Were Reduced by Enalapril Treatment

**Figure 2:**
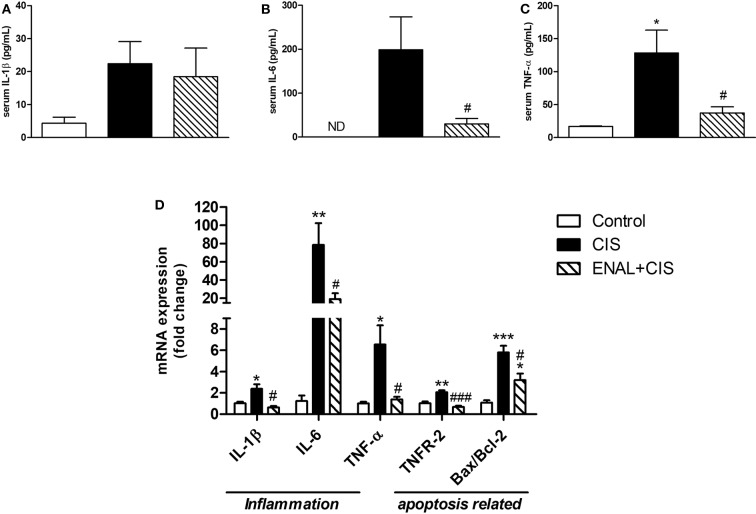
Analysis of serum levels of cytokines and kidney expression of cytokines and apoptotic markers mRNA. **(A–C)** serum levels of pro-inflammatory cytokines; **(D)** mRNA expression of cytokines and receptors involved in inflammation and mRNA expression ratio of pro- and anti-apoptotic proteins 4 days after cisplatin treatment. *p <0.05, **p <0.01, ***p <0.001. compared to the control group. #*p* <0.05; ###*p* < 0.001. compared to the CIS group. Data presented as Mean ± SEM *n* = 5–6.

Serum levels of some pro-inflammatory factors and markers, such as IL-1β, TNF-α, and IL-6 were measured. A reduction mainly in TNF levels was observed in the ENAL+CIS group when compared to the CIS group (Figures 2A–C). We also analyzed the renal expression of these factors and all of them presented reduced mRNA expression in the enalapril-treated group, when compared to the CIS group (Figure 2D). Preventive treatment with enalapril reduced the intrinsic pathway of apoptosis, as evidenced by the pro-apoptotic/anti-apoptotic ratio shown by Bax/Bcl-2 mRNA expression (Figure 2D), as well as the extrinsic pathway, as evidenced by TNFR-2 mRNA expression (Figure 2D). This result suggests a reduced signaling for apoptosis in mice treated with enalapril when compared to mice treated with cisplatin alone.

### Enhanced B1 Receptor mRNA Expression Was Inhibited by Enalapril Treatment

**Figure 3:**
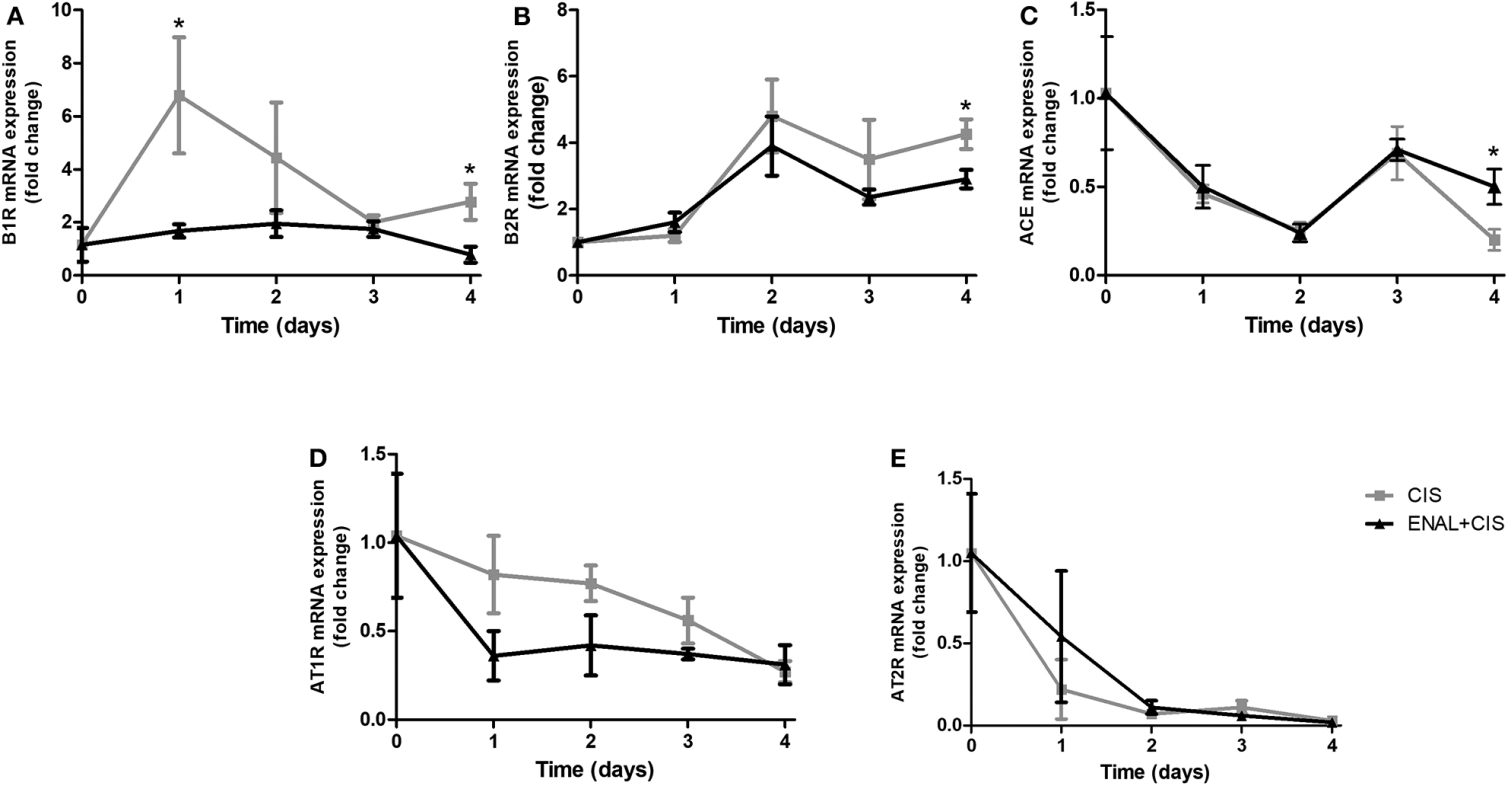
**(A–E)** Renal mRNA expression of RAS and KKS receptors and ACE. The panels show the daily renal mRNA expression after cisplatin injection. B1R and B2R = B1 and B2 kinin receptors; AT1R and AT2R = AT1 and AT2 angiotensin II receptors. *p <0.05. Data presented as Mean ± SEM of fold changes; *n* = 5–6 per group. CIS = Cisplatin; ENAL + CIS = Enalapril + Cisplatin.

Kinin B1 receptor is pro-inflammatory and its expression can be induced by inflammation. Enalapril treatment prevents the rise of B1 receptor mRNA expression (Figure 3A). Kinin B2 receptor also showed an increased expression after cisplatin injection, but the difference between cisplatin-treated groups was small and presented statistical difference only on the fourth day (Figure 3B). The expression of ACE was lower in ENAL + CIS group on the fourth day (Figure 3C). The mRNA expression of other renin-angiotensin system components, such as the AngII receptors, AT1R and AT2R, was not strongly affected by preventive treatment with enalapril (Figures 3D,E).

### Enalapril Treatment Restores Aminopeptidase P Activity

**Figure 4:**
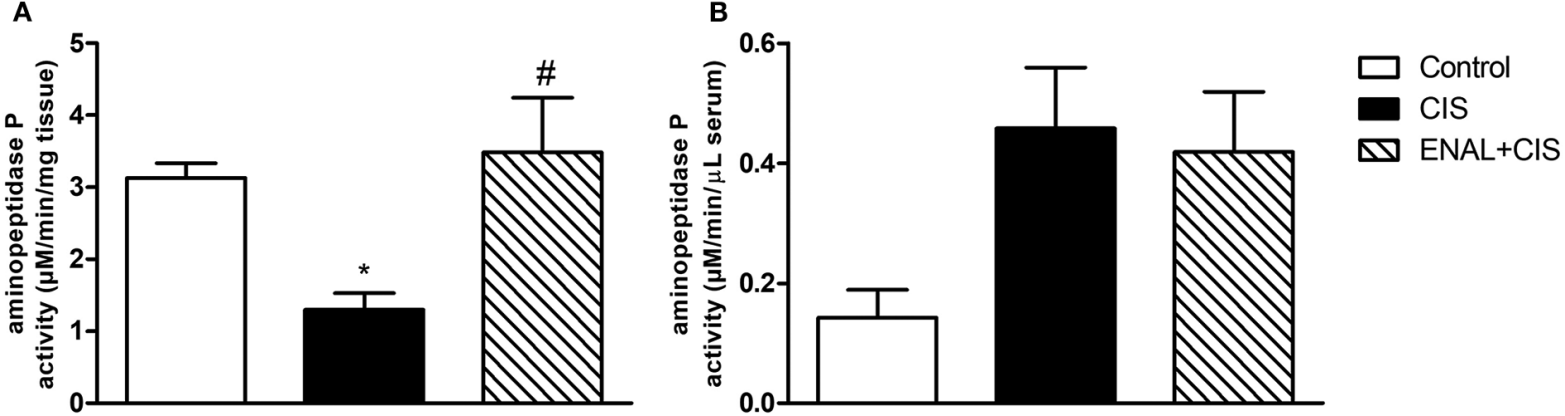
Renal and serum aminopeptidase P activity. Enalapril treatment prevents reduction in aminopeptidase P activity in kidney **(A)**, no alterations were found in aminopeptidase P activity in serum **(B)** 4 days after cisplatin injection. *p < 0.05 compared to the control group; #*p* < 0.05 compared to the CIS group. Data presented as Mean ± SEM; *n* = 6 per group.

In the presence of a strong ACE inhibitor, such as enalapril, the degradation of kinin peptides takes place by non-ACE pathways (Kim et al., 2000). Aminopeptidase P (APP) has a significant role in this degradation. Cisplatin reduces APP activity in the kidney, diminishing kinin degradation and increasing its inflammatory effects. On the other hand, enalapril treatment restores APP activity in the kidney (Figure 4A). No differences were found in serum APP activity after cisplatin treatment (Figure 4B).

### Enalaprilat Reduces Cisplatin-Induced B1R and Pro-inflammatory Cytokines in Mouse Epithelial Tubular Cells

**Figure 5:**
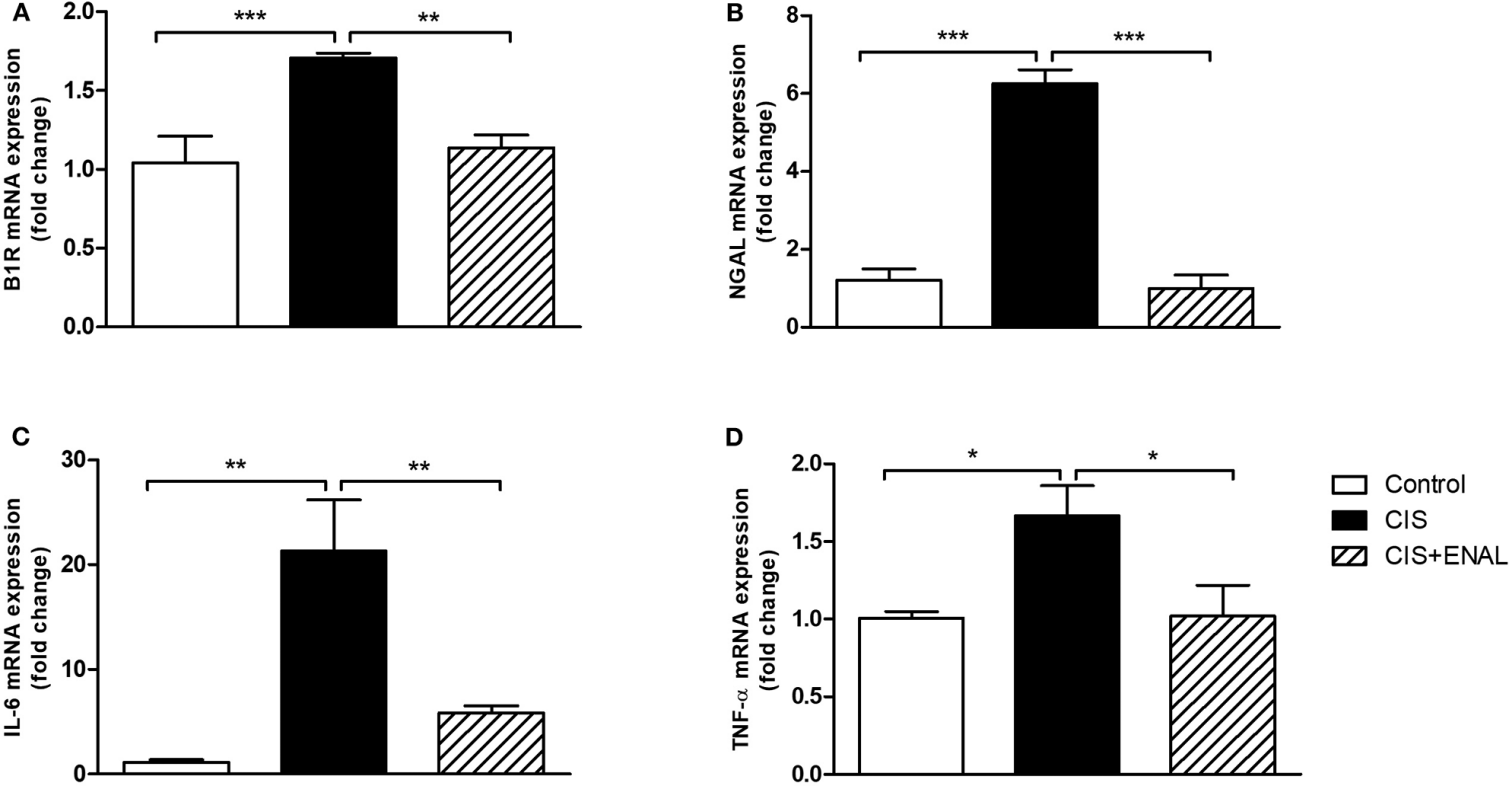
Mouse epithelial tubular cells mRNA expression. The panels show mouse tubular cell mRNA expression of **(A)** B1R, **(B)** NGAL, **(C)** IL-6, and **(D)** TNF-α after treatment with cisplatin and cisplatin + enalaprilat. Data presented as Mean ± SEM; *p < 0.05, **p < 0.01, ***p < 0.001.

In order to confirm the data found in animals, mouse epithelial tubular cells MK55.K were incubated with cisplatin and cisplatin+enalaprilat. Cisplatin increased B1R, NGAL, IL-6, and TNF-α mRNA levels. Enalaprilat was able to abolish the increase of these markers in mouse epithelial tubular cells (Figures 5A–D).

### Enalaprilat Attenuates Cisplatin Toxicity in Mouse Epithelial Tubular Cells and Apstatin Blocks Its Effects

**Figure 6:**
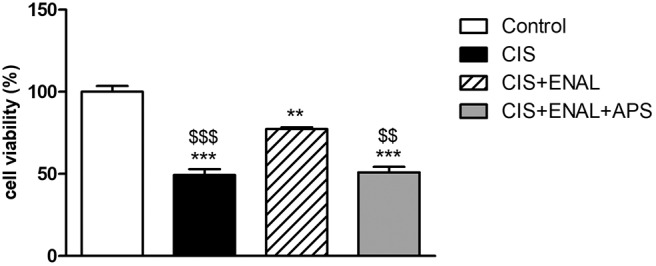
Cell viability assay in mouse epithelial tubular cells. The panels show cell viability assay in mouse tubular cells after treatment with cisplatin, enalaprilat and apstatin. Data presented as Mean ± SEM; **p < 0.01, ***p < 0.001 compared to the control group. $$*p* < 0.01 $$$*p* < 0.001 compared to the CIS+ENAL group.

In this study, we treated mouse epithelial tubular cells MK55.K with cisplatin, enalaprilat and apstatin (APP antagonist). Cisplatin decreased cell viability, while enalaprilat was capable of attenuating this decrease. Moreover, apstatin combined with cisplatin and enalaprilat blocked enalaprilat benefits in mouse epithelial tubular cells (Figure 6).

### ACE Inhibition Blunts Increase of Carboxypeptidase M Expression *in vivo* and *in vitro* After Cisplatin Treatment

**Figure 7:**
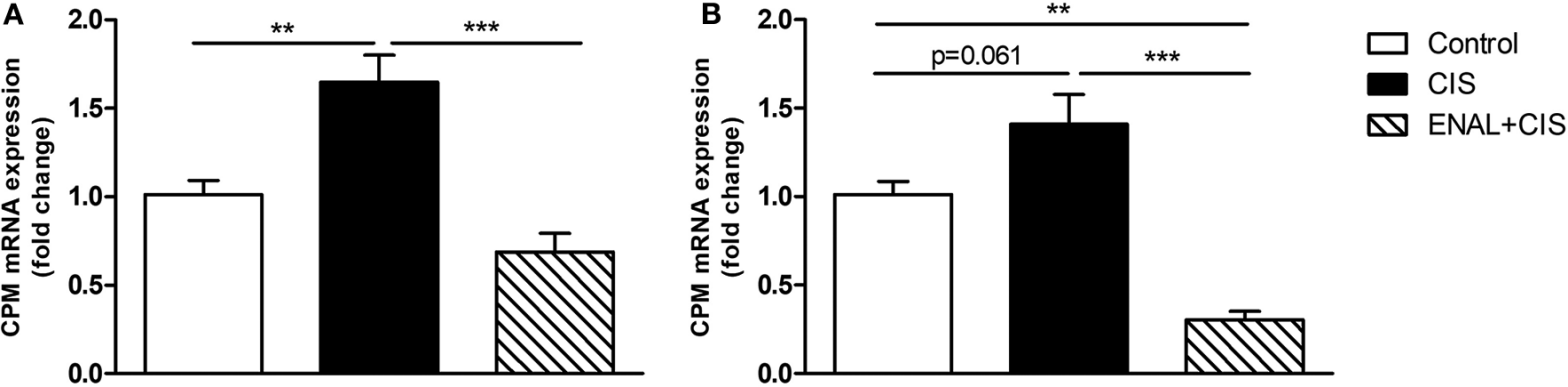
Carboxypeptidase M mRNA expression in renal tissue and mouse epithelial tubular cells. The panels show CPM mRNA expression **(A)** in mice 24 h after treatment with cisplatin or cisplatin plus enalapril and CPM mRNA expression **(B)** in mouse tubular cells after treatment with cisplatin or enalaprilat plus cisplatin. Data presented as Mean ± SEM; **p < 0.01, ***p < 0.001.

Carboxypeptidase M (CPM) facilitates B1R signaling (Zhang et al., 2008, 2011, 2013a,b; Guimarães et al., 2019). The peak of B1R expression after cisplatin injection is at 24 h; therefore, we analyzed CPM expression in renal tissue 24 h after cisplatin treatment. CIS increased CPM expression while ENAL+CIS blunted this effect (Figure 7A). Mouse epithelial tubular cells were treated with 50 μM CIS, which increased B1R expression. We observed also a trend to increased CPM expression, while ENAL+CIS largely reduced its expression (Figure 7B).

## Discussion

The use of cisplatin in chemotherapy bears the risk of renal injury. This is the main limitation for the use of this drug in the treatment of some types of tumors. Preventing these damaging effects may grant the use of higher doses of cisplatin and improve its effect as a chemotherapeutic drug. Previous studies have shown that the blockage of kinin B1 or B2 receptors could protect the kidney from cisplatin-induced injury in mice (Estrela et al., 2014a,b). These results open the possibility of using B1 and B2 receptors antagonists for preventing renal injury in patients undergoing cisplatin treatment. These receptors are activated by kinins, which are mainly degraded by ACE. ACE inhibitors reduce kinin degradation, are low-cost drugs and have widespread use. They have few side effects and are approved for human use. In the present work, we tested the effect of an ACE inhibitor and found that enalapril treatment can also protect the kidney from cisplatin-induced injury in mice. This conclusion was drawn based on its ability to reduce cytokines expression, reduce serum TNF-α, urea and creatinine levels, and to reduce tubular injury and apoptosis signaling, as shown in mice treated with cisplatin + enalapril when compared to animals treated with cisplatin alone.

Since enalapril inhibits ACE and consequently leads to the increase of kinin peptides' half-life, we expected an increase in deleterious effects of cisplatin in the kidney. Nonetheless, the results indicated the opposite.

We analyzed both KKS and RAS components in an attempt to understand the mechanisms involved in this protective effect. Interestingly, although an ACE inhibitor drug was used, only small changes were found in mRNA expression of the main RAS components, such as AT1R, AT2R or ACE. One of these changes was noted in ACE mRNA expression, which was lower in ENAL+CIS group on the fourth day. On the other hand, a strong effect was observed in KKS expression, as noted by enalapril effect of blocking the increased expression of B1R caused by cisplatin injection. Therefore, we confirmed, *in vivo*, the phenomenon first observed *in vitro* by Ignjacev-Lazich et al. (2005), i.e., the effect of ACE on the expression of B1R and B2R genes. These authors showed that increasing ACE expression in the vascular smooth muscle cell culture strongly induces the upregulation of B1R and B2R expression by 22- and 11-fold, respectively. This effect was inhibited by captopril, but not by AT1R or AT2R antagonists (Ignjacev-Lazich et al., 2005). In the present study, we showed that the inhibition of ACE activity also downregulates the expression of both kinin receptors, with a more pronounced effect on B1R expression. Given that kinin peptides have a short half-life in the organism and that other enzymes are able to degrade kinins, we speculate that the reduced expression of kinin-receptor genes has a stronger inhibitory effect in KKS, overcoming the possible increase in kinin concentration or half-life. This data strongly suggests a mechanism for the protective effect observed by enalapril treatment on cisplatin-induced renal injury.

The blockage of both B2R and B1R is able to reduce cisplatin-induced renal injury, as observed before (Estrela et al., 2014a,b). The established knowledge that B2R is constitutively expressed in most tissues including the kidney, and that B1R expression is inducible by inflammation (Leeb-Lundberg et al., 2005) may explain why only the B1R expression was strongly affected by the preventive treatment with enalapril, while the B2R expression presented only a smaller change. On the other hand, it is possible that the reduction in B1R expression could be secondary to lower inflammation caused by enalapril effects on other biological targets, such as the decrease of AngII production. Moreover, it has been shown that AT1R activation by AngII directly increases B1R expression (Parekh et al., 2019). AngII is capable of inducing kinin B1R via AT1R by releasing endothelin-1 and activating the endothelin receptor A (Morand-Contant et al., 2010). Moreover AngII can induce B1R through AT1R also by activating NF-κB (Fernandes et al., 2006). B1R is capable of increasing the production of iNOS and NADPH oxidase, which leads to increased production of peroxynitrite and superoxide anion enhancing oxidative stress and activating NF-κB and the transcription of several inflammatory cytokines such as IL-1β and TNF-α, iNOS, and B1R (Othman et al., 2019). Hence, the inhibition of B1R signaling with enalapril can account for the decrease production of these inflammatory cytokines in our study.

Since upregulation of B2R by cisplatin is even more sustained and synergistically contributes to renal damage (Estrela et al., 2014b), the slight but significant reduction in B2R expression by enalapril may also be of therapeutic relevance.

In the present study, while no strong changes in renal AT1R expression were observed in enalapril-treated mice, part of the reduction in renal inflammation and damage can also be due to the expected reduction of AngII peptide, since enalapril is a strong ACE inhibitor. AngII has been associated to kidney inflammation in several models (Theuer et al., 2002; Altunoluk et al., 2006; Benigni et al., 2011; Nagasawa et al., 2012; Kanda et al., 2016; Ham et al., 2018; Panico et al., 2019), and the reduction of its formation certainly has an anti-inflammatory effect. Therefore, the changes in KKS components must be considered mainly because they have been tested before alone (Estrela et al., 2014a,b), with no inhibition of AngII formation, and presented similar effects as those shown herein. The evidence indicates the participation of KKS regulation in the mechanism involved in renal protection against cisplatin side effects without discarding the additional effect via AngII formation inhibition.

The measurement of kinin concentration in mice kidney tissues is not feasible. However, it is possible to hypothesize that kinin half-life or concentration in the kidney of mice treated with both cisplatin and enalapril was increased. On the other hand, the increased expression of aminopeptidase P (APP) can be thoroughly discussed. APP is the second enzyme in rank to degrade bradykinin, responsible for 30% of bradykinin inactivation in the presence of active ACE (Prechel et al., 1995; Fryer et al., 2008). This is the main non-ACE pathway of kinin degradation and we speculate that the observed effect of enalapril in restoring APP activity in the kidney must increase kinin degradation by this non-ACE pathway, thus reducing inflammation (Kim et al., 2000; Fryer et al., 2008). Moreover, we confirmed the importance of APP in cultured cells, in which the combined treatment of cisplatin plus enalaprilat attenuated cisplatin toxicity in renal epithelial tubular cells. When we combined cisplatin, enalaprilat and apstatin, cell viability was decreased, showing the importance of APP on enalaprilat protective effects.

Carboxypeptidase M (CPM) is an ectoenzyme (Zhang et al., 2013b), its catalytic domain hydrolyzes Lys or Arg from C-terminal peptides (i.e., kallidin, bradykinin) which in turn generates B1R agonists, des-arg10-kallidin or des-arg9-BK (Skidgel et al., 1989). Moreover, CPM enhances B1R signaling (Zhang et al., 2008, 2011, 2013a,b; Guimarães et al., 2019). In our study we showed that B1R expression was diminished by enalapril and enalaprilat treatment both in mice and cells and that the generation of B1R agonists is also reduced due to a decrease in CPM expression *in vivo* and *in vitro*.

**Figure 8:**
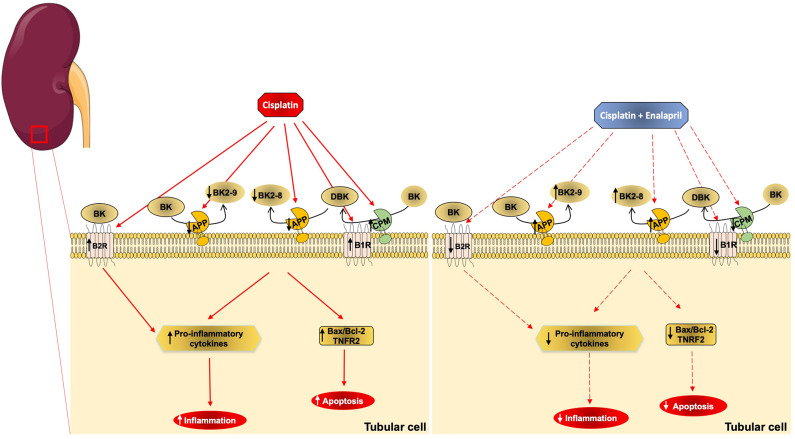
Schematic representation of cisplatin and enalapril effects on aminopeptidase P and B1R signaling in renal tubular cells. The scheme shows that cisplatin (CIS) decreases APP activity, which leads to decreased kinin degradation that increases des-arg9-BK (DBK) availability and leads to increased inflammation and apoptosis. It also shows that B1 receptor and CPM upregulation by cisplatin toxicity increases B1 receptor signaling. Additionally, it shows that enalapril+CIS increases APP activity compared to CIS alone, increasing kinin degradation, which decreases des-arg9-BK (DBK) concentration, lowering inflammation and apoptosis. On the other hand, enalapril also decreases B1 receptor and CPM expression reducing B1 receptor signaling.

Taken together, the results suggest that the protective effect of enalapril on cisplatin-induced renal injury is not only due to a reduction in AngII formation, but also by the modulation of KKS components, mainly by blunting CPM expression, thus reducing kinin B1 receptor agonism and its overexpression, and preventing increases in kinin peptide concentrations through the increased APP activity, acting locally in renal epithelial tubular cells (Figure 8).

## Data Availability Statement

All datasets generated for this study are included in the article/supplementary material.

## Ethics Statement

The animal study was reviewed and approved by The animal study was reviewed and approved by Comitê de ética no uso de animais (CEUA) from Universidade Federal de São Paulo (UNIFESP).

## Author Contributions

GE, NC, JP, MB, CB, and RA designed the study. GE, FW, AA, MG, RM, and DM performed the experiments. GE, RA, MB, DM, and LF-L analyzed the data. GE, LF-L, CB, and RA wrote the paper.

## Conflict of Interest

The authors declare that the research was conducted in the absence of any commercial or financial relationships that could be construed as a potential conflict of interest.
